# Ultrasound-Guided Needle Aspiration vs. Surgical Incision of Parotid Abscesses

**DOI:** 10.3390/jcm11247425

**Published:** 2022-12-14

**Authors:** Ulrich Strassen, Christophe Grimler, Benedikt Hofauer

**Affiliations:** Department of Otorhinolaryngology/Head and Neck Surgery, Technical University of Munich, 81675 Munich, Germany

**Keywords:** parotid gland, abscess, surgical drainage, ultrasound, needle aspiration

## Abstract

Objective: Standard treatment of parotideal abscesses consists of surgical drainage. This often has to be carried out in general anesthesia and carries the risk of iatrogenic injury of the facial nerve. Ultrasound-guided needle aspiration is an alternative therapy. Up until now a lack of systematic data concerning this subject exists. The study at hand aims to answer the question whether needle aspiration is a viable alternative for surgical drainage. Methods: All patients who had been treated surgically (*n* = 39) or via ultrasound-guided needle aspiration (*n* = 18) at our clinic were included into this monocentric retrospective analysis. Results: There was no statistically significant difference (*p* = 0.142) regarding the mean abscess volume in both groups (5.7 vs. 10.1 mL). Therapy of the abscesses on average required 1.88 (1–5) ultrasound-guided needle aspirations or 1.10 (1–4) surgical interventions. There was a trend to a shorter inpatient treatment period (5.88 vs. 7.33 days) after ultrasound-guided needle aspiration. This trend did not reach statistical significance (*p* = 0.301). Facial nerve alterations did not occur in any of the patients. Postoperative bleeding did never occur after needle aspirations but in 2% of the patients after surgical abscess revision. Conclusion: Ultrasound-guided needle aspiration is safe and effective in the treatment of parotid abscesses.

## 1. Introduction

Even if the parotid gland is the largest salivary gland and most frequently affected by inflammation, parotitis rarely develops into an abscess [1,2]. Etiologically, a parotid abscess can be attributed to bacterial, viral, obstructive, or immunological causes, among others. In adults, the triggers are more often of bacterial than viral origin (e.g., HIV-associated) [1,3]. In the case of an enlarged parotid gland, numerous other possible etiologies must be considered, including sialolithiasis, sialadenitis, chronic recurrent parotitis, cystic fibrosis, vascular diseases, alcoholism, or neoplasms [3,4,5]. The proportion of neoplasms originating from the salivary glands is given as around 3%, with the majority having its origin in the parotid gland [6]. The advantage of needle aspiration-based approach in such cases is that the risk of a tumor opening and seeding is largely avoided and a one-stage R0 resection can be carried out if there is cytological evidence of the tumor.

Although parotid abscess often occurs in slightly advanced age (>50 years) and in immunocompromised individuals, it can affect all age groups. Since parotid abscesses potentially involve deep neck areas and can result in systemic infections, serious complications may occur ranging from facial nerve palsy, osteomyelitis of the jaw to temporal lobe abscess, descending mediastinitis, septicemia, for example [2,7].

If there is no adequate improvement with conservative treatment modalities, additional/or invasive therapy options such as surgical incision and drainage has to be considered. The classic surgical procedure is open incision and drainage, using either a standard parotidectomy or a transcervical approach [1,8]. Potential disadvantages include the need for general anesthesia if neuromonitoring is required or due to pain-related lack of patient compliance, the long incisions that may be needed or the sometimes extensive dissection, and the associated risk of injury to the facial nerve, but also potentially unfavorable cosmetic outcomes from scarring [8,9].

Ultrasonography-guided needle aspiration of parotid abscess offers a less invasive alternative to surgical incision and drainage. A sonographic approach is considered to be a diagnostic tool of high reliability and the imaging method of choice for diagnostic evaluation of the parotid gland, but it also opens up possible advantages in the context of abscess treatment. These can include faster healing, shorter hospitalization times, or better cosmetic results [3,8,10,11].

To date, there are no evidence-based, unitary recommendations as to which treatment approach is to be preferred. Experience to date is primarily based on retrospectively collected case reports or series, and the number of cases is therefore limited. However, the few available observations suggest that ultrasound-based needle aspiration is generally associated with fewer complications than surgical incision or drainage—with comparable success rates [12,13]. According to a recent systematic review, 18 total patient cases of parotid abscesses treated by “ultrasound-guided aspiration” have been reported in the published literature between 1999 and 2021 without any complications [12]. In light of the lack of systematically collected data, we performed a comprehensive retrospective evaluation of all patient cases who underwent surgical treatment or ultrasound-guided fine-needle aspiration at our clinic for parotid abscess during a period of eleven years.

The primary goal of the study was therefore to determine whether needle-aspiration is a viable option to surgical drainage in terms of complication rates, length of stay in hospital, and the frequency of required interventions. The secondary goal consisted of the determination of causes of parotideal abscesses (including the microbiological spectrum of pathogens) and the clinical presentation of the disease.

## 2. Material and Methods

We retrospectively reviewed all available medical records on patients undergoing treatment for a parotid abscess from January 2008 to December 2019 at the department of otolaryngology/head and neck surgery, university hospital of the Technical University of Munich (TUM). Diagnostic work-up of all patients consisted of inflammatory markers (leucocytes and CRP) and B-mode and color-duplex sonography by the head-and-neck surgeon later performing the puncture or incision. CT scans were routinely performed in all patients before open surgical drainage. All patients received a follow-up visit including sonographic re-evaluation one month after discharge from the clinic. The evaluated treatment period of this monocentric study covers a total experience period of eleven years.

All cases of parotid abscess undergoing surgical treatment or ultrasound-guided needle aspiration (20 gauge needle) were included in this study. Surgical drainage was carried out either in local or general anesthesia (patients’ choice). Surgical incisions (1 to 3 cm) were placed superficially to the sonographically identified abscess and carried out parallel to the estimated course of the relevant facial nerve branches. The abscess cavity was then identified and opened via blunt dissection. Easy flow drainages were subsequently introduced. Drainages were removed after putrid secretion had seized.

Excluded from analysis were abscesses not involving the parotid gland or managed by conservative treatment alone (*n* = 3). Conservative treatment consisted of Ampicillin/Sulbactam 1.5 g three times daily for a period of 10 days. In addition to information on patient characteristics such as age, sex, presumed disease etiology, or comorbidities, data on the abscess of the parotid gland such as imaging approach at diagnosis, localization and extent, clinical symptoms, therapy outcome (duration of hospitalization, duration of symptoms up until hospital admission, number of interventions, complications) or microbiological findings were collected, if available on medical records. The data were analyzed on a descriptive basis.

Patients that had to be readmitted to the hospital with a parotid abscess on the same side were considered as recurrences. In these cases, the depicted duration of the hospital stay represents only the first stay.

When patients failed to improve clinically or inflammatory values (CRP, leucocytes) kept rising after FNP, this was considered as a therapeutic failure. These patients were treated via surgical drainage.

## 3. Results

### 3.1. Patient Characteristics

A total of 57 patients with parotid abscess were identified, one patient was treated with a second parotid abscess on the opposite side during a second hospital stay, resulting in a total of 58 cases. These patients received either ultrasound-guided needle aspiration (*n* = 18) and/or surgical incision therapy (*n* = 40). Patients’ average age was 56.12 years (range: 17–90 years). A proportion of 56.14% (32/57) of all patients was female. In the needle aspiration group, the case-related gender ratio was 11:7 for females and males, and 22:18 for the surgical treatment group. As summarized in Table 1, hypertension and type 2 diabetes mellitus were among the most common pre-existing comorbid diseases. Also listed are the available data on abscess-related symptoms and laboratory parameters (c-reactive protein, leukocytes) at baseline, and imaging techniques used in diagnostic evaluation. Regarding etiology, the underlying cause of abscess formation was documented in 5 of 18 cases needle aspiration group, but in 72.22% unknown (13/18). In the surgical incision group, the cause of the parotideal abscess was recorded in 10 of 40 cases, and here the presumed cause was unknown in 75% (30/40). In three cases, a parotitis could be identified as the cause, a further three cases were based on a Sjogren’s syndrome. In one case, the abscess formation was due to MALT lymphoma. Other diagnoses, each identified as a possible cause (*n* = 1), included a case of lateral parotidectomy, chronic sialadenitis, pyoderma gangrenosum, lymphadenitis, superinfected cystic mass, Warthin’s tumor/suspicion of neoplasia, and iatrogenic injury (Table 2).

Abscess lesions were located equally on the right and left side (*n* = 28), two lesions were bilateral (Table 2). Limitations of this study included the retrospective data review and small number of cases.

### 3.2. Treatment Outcomes

In four of 58 cases, both therapy modalities were used during one same hospital stay. In three cases, patients were treated surgically because fine-needle aspiration did not lead to a sufficient improvement of symptoms, and in one case the patient was treated by fine-needle aspiration after the surgical exploration failed to identify the abscess. Seven patients had abscess recurrence requiring a second hospitalization plus intervention: The parotid abscess recurrence was treated again by surgical incision (*n* = 7). The mean pus-drained volume was 5.7 mL (median: 4.5; range: 0.5–16.6) in the fine-needle aspiration group and 10.1 mL (median: 7.7; range: 1.2–36.9) in the surgical group. The abscess volumes were subsequently calculated from the available information on abscess length, width, and height ($\frac{4\pi }{3}\times \frac{length}{2}\times \frac{width}{2}\times \frac{height}{2}$).

The mean length of hospital stay was 5.88 days (median: 7; range: 0–12) in the ultrasound-guided aspiration group and 7.33 days (median: 8; range: 2–13) in the surgical group, in case of recurrences only the first hospital stay was included. The abscess-related symptoms had lasted 4.29 days (median: 4; range: 1–7) on average up until hospital admission in the fine-needle aspiration group, with patients in the surgical treatment group remaining symptomatic slightly longer with an average of 11.36 days (median: 7; range: 1–56) (Table 3). No statistically significant group differences (*p* = 0.272) could be shown regarding age.

There were eight cases of multiple punctures in the aspiration group. In six cases, a second puncture was required, in one case five punctures were performed on consecutive days. In the surgical group, revision was required in 2 cases (in the first case a second incision, in the other case a needle aspiration after a previous incision). On average, 1.88 interventions (median: 2; range: 1–5) were required in the fine-needle aspiration group, and 1.10 interventions (median: 1; range: 1–2) in the surgical group (Table 3). Facial paralysis did not occur as a complication in either the surgical treatment group or the aspiration treatment group. There were also no salivary fistulas, which can occur as an iatrogenic complication, especially with an incision. Bleeding was reported only in the surgical group (*n* = 1); recurrences were reported in the surgical group in four cases, and in three cases in the fine-needle aspiration group. One patient in the fine-needle aspiration group presenting with a recurrence had been treated surgically due to fine-needle aspiration treatment failure during the first hospital stay and had a second recurrence that was again treated surgically. This patient later presented with a third recurrence that was treated by parotidectomy. One patient from the surgical group, who required a second incision and later presented a recurrence was treated surgically and at a later date underwent a parotidectomy.

The most common organisms grown from bacterial culture results were *Staphylococcus aureus* (*n* = 10), followed by *Streptococcus intermedius* (*n* = 7) and *greening Streptococci* (*n* = 6) (Table 4). No bacterial growth from cultures was found in three abscesses from the ultrasound-guided fine-needle aspiration group and from nine abscesses in surgical group.

## 4. Discussion

Management of parotid abscesses includes conservative antibiotic treatment and/or surgical incision and drainage, imaging-guided percutaneous drainage, or needle aspiration. The treatment decision is usually based on the individual preferences of the clinician [12]. Fully published descriptive or controlled studies on the question of whether ultrasound-controlled needle aspiration is a safe and effective alternative to the surgical procedure are still lacking. So far, there are also no clearly defined criteria, in which cases the needle is most likely to be used and in which cases certain patients (e.g., smaller abscesses with less pronounced swelling?) are most likely to benefit [8]. To our knowledge, the present analysis represents the data collection with the highest total case number of parotid abscesses. Preliminary data indicate that ultrasound-guided needle aspiration is not inferior to surgical incision and drainage while at the same time reducing the risk of complications [12,13]. Ultrasound-guided needle aspiration can be performed repeatedly without increased risk of exposure to radiation, frequent bleeding, irreversible injuries of neurovascular structures, or harmful effects by general anesthesia [9,14]. In addition, the use of the contrast-enhanced ultrasound has the advantage that it enables the differentiation between neoplastic tissue and avascular abscess formation within the parotid gland, or between vascularized and non-vascularized areas [9].

Within our patient population, a bleeding complication occurred in only one case, which affected the surgical group. Repeated needle aspiration (even over several days) did not result in any bleeding or injury-related complications. With a hospital stay of on average 5.88 days, treatment cases that were carried out under ultrasound monitoring required a slightly shorter inpatient stay than surgically treated cases (average 7.33 days). The median length of stay was 7 days in the fine-needle aspiration group and 8 days in the surgical group. Symptom duration was also, on average, shorter in patients who underwent aspiration than in patients in the surgical group (4.29 vs. 11.36 days). Pain, swelling, redness or localized hyperthermia were reported more frequently in surgically treated patients overall than in patients in the aspiration group.

From a randomized controlled comparative study with a total of 32 patients who underwent ultrasound-guided aspiration vs. drainage for deep neck abscesses, only the poster of an initial evaluation is available so far. The study authors concluded that ultrasound-guided aspiration offers a safe and effective alternative to surgical incision and drainage for “uni- or multilocular deep neck abscesses”. In addition, aspiration was associated with a shorter hospital stay (2.63 vs. 4.81 days) [13]. It has to be added though that ultrasound-guided aspiration was not sufficient as a treatment option in 16.6% (*n* = 3) of the patients in our study. Those patients had to undergo subsequent surgical drainage. According to a previous study, shorter hospital stays for ultrasound-guided, less invasive drainage of deep neck abscesses may also be associated with significant cost savings versus longer hospital stays for surgical drainage [15]. A corresponding cost comparison between the ultrasound-based aspiration procedure and the surgical intervention—even if aspiration was repeated two (*n* = 3) or five (*n* = 1) times in individual patients—could come to a similar result.

Since the ultrasound-determined measurement parameters were not available for every patient, the volume calculation of an abscess could only be carried out in 9 of 18 cases during needle aspiration, or in 29 out of 40 surgically treated cases. Despite these limitations, the results could indicate a trend toward higher abscess volumes in the surgical group (10.1 vs. 5.7 mL). Consistent with this, patients undergoing surgical incision generally appeared to have a higher symptom burden at baseline than patients treated with aspiration—particularly in terms of pain, swelling, and redness of the lesions. It is therefore possible that treating physicians chose surgical incision as a therapeutic option in larger abscesses leading to a bias in the presented data.

Women were slightly overrepresented in both groups, the gender ratio was 11:7 for ultrasound-based needle aspiration and 22:18 for surgical incision. On average, patients in the ultrasound-guided needle aspiration group were slightly younger than in the surgical group (51.78 vs. 58.68 years). This trend did not reach statistical significance. A possible explanation for this fact might be the wish of younger patients to avoid scaring and the (though very small) risk of a lesion of the facial nerve as a consequence of surgical drainage. The mean age of all patients (56.12 years) in our patient population was in general consistent with the results of other retrospective data collections on parotid abscesses in adults [12,16,17]. In accordance with the literature [1,12,17], *Staphylococcus aureus* was also one of the most frequently detected bacterial pathogens in our data collection. *Streptococcus intermedius* (7 cases) and *greening Streptococci* (6 cases) were mentioned less frequently in the more recent literature as pathogens associated with parotid abscesses [12], but are already listed as possible pathogens in earlier reviews [4,18]. According to literature [4,15], the lacking evidence of bacterial growth (11 cases in this study) seems also to be a common finding and is frequently observed in chronically recurrent sialadenitis [4]. Sialadenitis has been reported as one of the most common causes of parotid abscesses, along with odontogenic causes [19]. In our evaluation, chronic sialadenitis was documented in at least one case as the probable cause.

## 5. Conclusions

Ultrasound-guided needle aspiration offers a safe and effective treatment approach for parotid abscesses. Prospective studies with statistically sufficient numbers of cases comparing ultrasound-guided needle aspiration and conventional surgical drainage as treatment modalities for parotid abscesses should be carried out to determine whether one of the two modalities shows more favorable results regarding the clinical patient outcome.

## Figures and Tables

**Table 1:** Patient characteristics.

Patient Characteristics	Ultrasound-Guided Fine-Needle Aspiration	Surgical Incision	Total
**Number of patients**	18	39	57
**Number of cases**	18	40	58
**Mean age in years (range)**	51.78 (17–81) *	58.68 (22–90) *	56.12 (17–90) **
**Gender ratio (female:male)**	11:7 *	22:18 *	32:25 **
Number of patients with the following comorbidities (*n*) *** *Arterial hypertension* *Type 2 diabetes mellitus* *Struma (multi)nodosa* *Sialolithiasis* *(Suspicion of) Warthin tumor* *Depression*	4 4 2 1 1 1	11 5 3 2 2 2	15 9 5 3 3 3
Number of following symptoms at presentation, case-related (*n*) *** *Pain* *Swelling* *Redness* *Local hyperthermia* *Trismus* *Dysphagia* *Fever* *Xerostomia* *Facial nerve palsy* *Pus in oral cavity* *Putride taste*	14 18 1 1 1 2 0 0 1 0 1	35 38 22 8 3 1 2 2 0 1 0	49 56 22 9 4 3 2 2 1 1 1
**Laboratory data** *CRP (mg/dL)* *Leukocytes (G/L)*	3.84 11.25	7.29 12.29	6.29 11.99
Diagnostic imaging (case-related) *** *Sonography* *Computed tomography* *Magnetic resonance imaging*	15 3 1	22 24 2	37 27 3

* case-related; ** patient-related, *** several entries possible per case.

**Table 2:** Abscess location and presumed cause of parotid abscess (where available).

	Ultrasound-Guided Fine-Needle Aspiration	Surgical Incision
**Abscess location (*n*)** *Right side* *Left side* *Bilateral*	7 11 0	21 17 2
**Presumed cause (*n*)** *Unknown* *Sjögren’s syndrome* *Marantic parotitis* *Purulent parotitis* *Chronic sialadenitis* *Lateral parotidectomy* *Pyoderma gangrenosum* *Lymphadenitis* *MALT lymphoma* *Cystic mass* *Warthin’s tumor* *Suspicion of neoplasia* *Iatrogenic (biopsy)*	13 1 0 1 0 1 0 0 0 1 0 1 0	30 2 2 0 1 0 1 1 1 0 1 0 1

**Table 3:** Treatment outcomes: differences between ultrasound-guided needle aspiration and surgical incision.

Parameter	Ultrasound-Guided Fine-Needle Aspiration	Surgical Incision	*p*-Value
Mean abscess volume in ml Median abscess volume (Range) in ml	5.7 4.5 (0.5–16.6)	10.1 7.7 (1.2–36.9)	0.244
Mean number of interventions, *n* Median number of interventions, *n* (range)	1.88 2 (1–5)	1.10 1 (1–2)	<0.01
Mean hospital stay in days Median hospital stay (range) in days	5.88 7 (0–12)	7.33 8 (2–13)	0.301
Mean duration of symptoms in days Median duration of symptoms (range) in days	4.29 4 (1–7)	11.36 7 (1–56)	0.02
**Complications** *Patients with abscess recurrence (n)* *Patients with bleeding (n)* *No pus punctured (n)* *Swap to surgical therapy due to FNP failure (n)*	3 0 1 3	4 1 0 1	0.175

**Table 4:** Microbiological results (where available) in patients undergoing ultrasound-guided needle aspiration and surgical incision.

Bacteria	Ultrasound-Guided Fine-Needle Aspiration	Surgical Incision	Total
*No bacterial growth*	3	9	12
*Streptococcus intermedius*	4	3	7
*Eikenella corrodens*	1	1	2
*Haemophilus parainfluenzae*	1	3	4
*Staphylococcus epidemis*	1	1	2
*Streptococcus mitis*	1	0	1
*Streptococcus tigurinus*	1	0	1
*Streptococus anginosus*	0	3	3
*Streptococcus constellatus*	0	2	2
*Finegoldia magna*	1	1	2
*Staphylococcus aureus*	2	8	10
*Greening streptococci*	2	4	6
*Parvimonas micra*	0	1	1
*Aggregatibacter aphrophilus*	0	1	1
*Candida glabrata*	0	1	1
*Streptococcus mutans*	1	0	1
*Neisseria macacae*	1	0	1
*Coagulase-negative Staphylococci*	1	1	2
*Haemophilus influenzae*	0	1	1
*Gemella sanguinis*	0	1	1
*Group C streptococci*	0	2	2
*Klebsiella pneumoniae*	0	1	1
*Bacteroides*	1	0	1
*Granulicatella adiacens*	1	0	1
*Fusobacterium naviforme*	1	0	1
*Streptcoccus pneumoniae*	0	1	1
*Enterobacter gergoviae*	1	0	1
